# Identifying Hand Use and Hand Roles After Stroke Using Egocentric Video

**DOI:** 10.1109/JTEHM.2021.3072347

**Published:** 2021-04-09

**Authors:** Meng-Fen Tsai, Rosalie H. Wang, José Zariffa

**Affiliations:** 1 KITE, Toronto Rehabilitation Institute, University Health Network 7989 TorontoONM5G 2A2Canada; 2 Institute of Biomedical Engineering, University of Toronto 7938 TorontoONM5S 1A1Canada; 3 Department of Occupational Science and Occupational TherapyUniversity of Toronto 7938 TorontoONM5S 1A1Canada

**Keywords:** Computer vision, egocentric camera, hand function, outcome measures, stroke

## Abstract

Objective: Upper limb (UL) impairment impacts quality of life, but is common after stroke. UL function evaluated in the clinic may not reflect use in activities of daily living (ADLs) after stroke, and current approaches for assessment at home rely on self-report and lack details about hand function. Wrist-worn accelerometers have been applied to capture UL use, but also fail to reveal details of hand function. In response, a wearable system is proposed consisting of egocentric cameras combined with computer vision approaches, in order to identify hand use (hand-object interactions) and the role of the more-affected hand (as stabilizer or manipulator) in unconstrained environments. Methods: Nine stroke survivors recorded their performance of ADLs in a home simulation laboratory using an egocentric camera. Motion, hand shape, colour, and hand size change features were generated and fed into random forest classifiers to detect hand use and classify hand roles. Leave-one-subject-out cross-validation (LOSOCV) and leave-one-task-out cross-validation (LOTOCV) were used to evaluate the robustness of the algorithms. Results: LOSOCV and LOTOCV F1-scores for more-affected hand use were 0.64 ± 0.24 and 0.76 ± 0.23, respectively. For less-affected hands, LOSOCV and LOTOCV F1-scores were 0.72 ± 0.20 and 0.82 ± 0.22. F1-scores for hand role classification were 0.70 ± 0.19 and 0.68 ± 0.23 in the more-affected hand for LOSOCV and LOTOCV, respectively, and 0.59 ± 0.23 and 0.65 ± 0.28 in the less-affected hand. Conclusion: The results demonstrate the feasibility of predicting hand use and the hand roles of stroke survivors from egocentric videos.

## I. Introduction

Hemiplegia or hemiparesis is commonly experienced after stroke. Unilateral motor deficit on the contralateral side of the brain lesion leads to decreased quality of life. In particular, upper limb function is one of the determinants of quality of life and independence after stroke [1]. An estimated 65% of stroke survivors experience difficulties in their activities of daily living (ADLs) as a result of upper limb impairment, despite medication and rehabilitation [2]–[4]. Measuring the upper limb function of stroke survivors in their daily life is vital to quantifying the impact of new interventions and to designing personalized rehabilitation plans.

Clinical upper limb function assessments for stroke survivors can measure different domains within the International Classification of Functioning, Disability and Health (ICF) developed by the World Health Organization. In particular, the activity domain can be subdivided into capacity and performance [5]. Capacity is defined as what a person can do in a standardized environment, and reflects the performance that can be achieved under ideal conditions for a given individual. Performance is defined as what a person actually does in their usual environment. To date, most clinical upper limb function assessments of activity after stroke are carried out in a hospital or clinic, and thus focus on capacity. The only two clinical assessments that capture the performance of stroke survivors are self-reported questionnaires: the Motor Activity Log [6] and the Stroke Impact Scale [7]. Self-reported questionnaires are hampered by attention deficiency and memory loss, which are commonly affected after stroke [8]. A valid and reliable clinical assessment to capture the upper limb performance of stroke survivors at home is still lacking. As a result, evaluating the true effectiveness of an intervention is a major challenge.

The measurement of performance in addition to capacity is particularly important in light of the fact that stroke survivors tend to use their more-affected hand less in their usual environments. This phenomenon is known as learned nonuse: more-affected hand movement is more effortful, which over time reinforces the compensatory use of the less-affected hand [9]. Studies have shown that the use of the more-affected limb is low in hospital settings (except for rehabilitation training) [10] as well as in the community [11]. Furthermore, Rand and Eng reported that even when upper limb function was improved after rehabilitation, stroke survivors were still prone to predominantly use their less-affected sides [10]. These results demonstrate that hand function evaluated in the clinic (capacity), does not reflect hand use in daily life (performance).

In response to this challenge, the use of wearable technology has been proposed to capture upper limb movements in ADLs [11], [12]. Accelerometers are commonly applied to record the upper limb use of stroke survivors in unconstrained environments [10], [13]. Wrist-worn accelerometers record arm movement but do not directly reflect hand movements. Finger-worn accelerometers have more recently been investigated, but to date have mainly been used in well-defined tasks in a laboratory setting [14]–[16] and only applied to real living environments in a limited number of studies [12]. A magnetic sensor, the Manumeter, has also been used to measure hand use in ADLs, however the device readings were reported to be sensitive to metal objects, such as utensils or doorknobs, which are common household objects [17], [18]. Both IMU-based and magnetic sensors capture only body movements and do not directly provide contextual information that could identify functional use of the hands. Therefore, there is still a need for alternative wearable technologies to capture hand use in daily life.

An egocentric camera is a wearable device that records videos from a first-person view. This technology has previously been used for life-logging and leisure applications [19], [20]. Because egocentric videos provide a clear view of the user’s hands and the tasks being performed, they have significant potential to measure hand use in home environments. This approach would require automated video processing methods capable of extracting clinically relevant information from large amounts of video data.

Computer vision methods for the analysis of hand movements in egocentric videos have been the focus of a number of previous investigations [21], dealing with problems including hand detection [22], hand segmentation [23], object detection/identification [24], [25], hand posture estimation [26], [27], and activity recognition [28], [29]. In the context of rehabilitation, the hand use of individuals with cervical spinal cord injury (cSCI) was detected by applying computer vision to egocentric videos, with promising results [30]. However, to the best of our knowledge, no previous work has analyzed the hand function of stroke survivors using egocentric video. Given the different disease symptoms and movement compensation patterns between the unilateral and bilateral hand impairments of stroke and cSCI, respectively, it is not currently known whether a similar approach would be successful in measuring hand use after stroke. Thus, the first aim of the present study was to demonstrate the feasibility of quantifying the hand use of stroke survivors during ADLs, using computer vision methods applied to egocentric video.

An additional consideration specific to understanding the hand use of individuals with hemiparesis is the role that the more-affected hand plays in bimanual interactions. Two hand roles during bimanual ADLs were examined in this study: stabilizer and manipulator [31]. Manipulators require more in-hand coordination, involving fine movements between fingers (fine motor), compared to stabilizers, which keep a fixed contact area between the hand and object during an interaction. The role played by the more-affected hand in bimanual object interactions depends on the level of upper limb impairment and hand dominance [31]. Classifying hand roles may offer a better understanding of the level of hand function and ADL performance of stroke survivors in their living environment. The second aim of our study was therefore to classify the role played by the more-affected hand during bimanual interactions in egocentric video.

## II. Methods and Procedures

### A. Procedure

Individuals who experienced a stroke were invited to participate in this study, which was approved by the Research Ethics Board of the University Health Network. Each participant made two visits to a home simulation laboratory at the Toronto Rehabilitation Institute – University Health Network. During the first visit, informed consent was obtained and clinical upper limb assessments were carried out to ensure that the inclusion criteria were met. In the second visit, participants carried out a series of ADLs while recording egocentric videos. These videos were used to train and evaluate two classifiers: one to detect interactions between the hands and objects in the environment (hand-object interactions) in each video frame, and the second to classify the role of the more-affected hand in bimanual interactions. The definition of a hand-object interaction is the manipulation of an object by the hand(s) for a functional purpose. For example, a hand resting on a table or swinging during walking would not be considered to be interacting, since no object is manipulated by the hand. Within the context of interactions, the definition of a stabilizer is that the hand is statically in contact with an object without changing the contact area between the hand and the object. In contrast, a hand is deemed to be a manipulator when it moves an object with the contact area changing over time. The rationale for this problem formulation is that hand-object interaction detection can be used as the basis for metrics reflecting independent use of the hand in ADLs at home, whereas the hand role classification can provide more insight specifically into use or non-use of the more-affected hand. Specific procedures for each step of the study are detailed in the following sections.

### B. Study Sample

Nine individuals who had experienced a stroke participated in this study. The upper limb impairment status and the functional performance of the participants were evaluated using the Fugl-Meyer Assessment for Upper Extremity (FMA-UE) [32], the Action Research Arm Test (ARAT) [33], and the Motor Activity Log (MAL) [34]. The inclusion criteria of study participants were: 1) at least six months post-stroke; 2) self-reported impact of more-affected hand on ADLs; 3) impaired but not absent hand function, defined as a total ARAT score above 10 [35]; 4) Montreal Cognitive Assessment above 21, to avoid potential cognitive difficulties [36]; 5) no subluxation or significant pain when using upper limb; 6) no other neuromusculoskeletal disease affecting upper limb movements other than stroke.

### C. Video Data Collection Procedure

**FIGURE 1.**
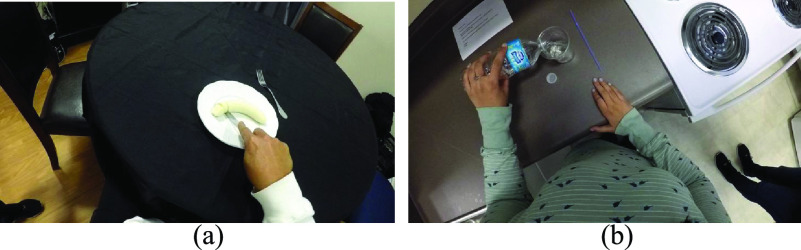
Instances of daily tasks performed by study participants. (a) Slicing a banana on a living room table (b) opening and pouring some water from a water bottle to a cup.

A head-mounted egocentric camera (GoPro Hero 5, GoPro Inc., San Mateo, CA, USA) was used to record how participants conducted 38 ADLs across six different room settings in the home simulation laboratory (see Supplementary Material). These included for example a food cutting task in a living room (Fig. 1 (a)) and a drink preparation task at a kitchen counter (Fig. 1 (b)). The participants were informed of what tasks to perform but not instructed on how to perform them. The egocentric videos were recorded at $1280\times 720$ resolution with 30 frames per second and analyzed at $640\times 360$ resolution. Portions of the videos were manually annotated frame-by-frame for interactions and hand roles according to the definitions above. A total of 81 tasks were chosen to be annotated. These varied between participants and included at least 6 tasks in different rooms from each participant in the home simulation laboratory. In addition, 2 tasks with no interactions per participant, consisting of each hand waving in the air, were also annotated and included to balance the dataset.

### D. Interaction Detection

**FIGURE 2.**
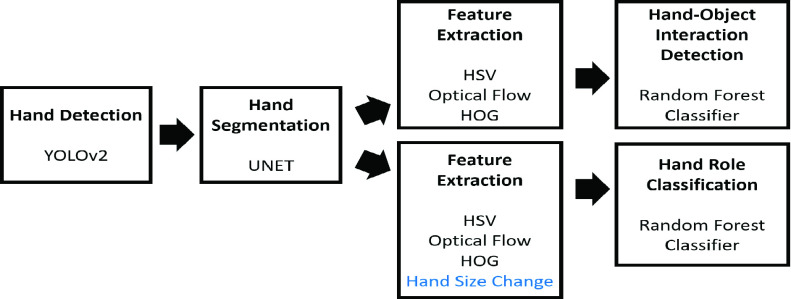
Analysis pipeline for hand-object interaction and hand role classification, with key methods for each step: hand detection, hand segmentation, feature extraction, interaction detection and hand role classification. (YOLOv2: You Only Look Once version 2; HSV: Hue, Saturation, and Value; HOG: Histograms of Oriented Gradients).

There were four steps to identify the hand-object interactions and hand roles from the egocentric videos: hand detection, hand segmentation, feature extraction and classification. The analysis pipeline is shown in Fig. 2.

#### a: Hand Detection

**FIGURE 3.**
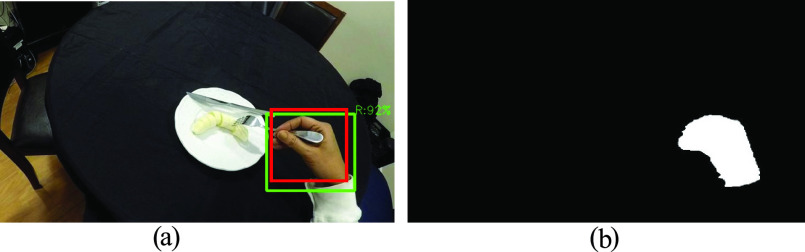
Hand detection and segmentation instances. (a) Hand bounding boxes (in green) were generated by YOLOv2 in each frame. The red box shows a manually labeled hand location. (b) The hands were segmented from the predicted (green) bounding boxes using UNET.

A convolutional neural network, You Only Look Once version 2 (YOLOv2) was trained in a previous study on individuals with cSCI to generate hand bounding boxes (green boxes in Fig. 3 (a)) in each egocentric frame [22]. The same network was applied here. Bounding boxes were manually labeled when at least 50% of the hand was visible in the frame. Evaluation was based on the Intersection over Union (IoU), defined as the overlapping area between the predicted and manually labeled bounding boxes (Fig. 3 (a) green and red box, respectively) over the total area of the combined boxes [37]. An IoU value closer to 1 denotes highly overlapping predicted and labeled hand locations. Here, an IoU value above 0.15 was considered a true positive, or otherwise, a false positive.

#### b: Hand Segmentation

A pre-trained U-shape convolutional neural network, UNET [38], [39], was trained on 4,080 frames from the EgoHands dataset [40] and 507 frames from our dataset to segment the two hands of the stroke survivors in our study. The generated hand segmentations (Fig. 3 (b)) within the bounding boxes were considered as hand regions and used for feature extraction.

#### c: Feature Extraction

**FIGURE 4.**
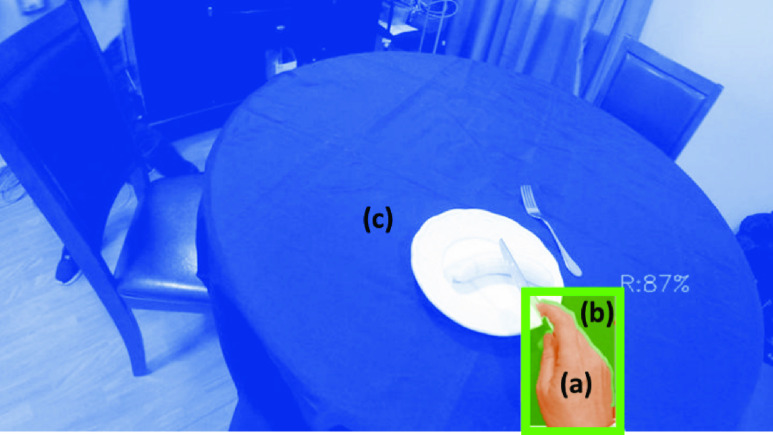
The diagram of three regions used in calculation of the colour and motion features: the hand region (a), the surrounding region (b) and the background (c).

Features based on colour, motion, and hand shape were extracted, based on previous work on interaction detection in cSCI [30]. Colour features used the Hue, Saturation and Value (HSV) colour space. The HSV histogram difference was calculated between the hand region (Fig. 4 (a)) and the bounding box area surrounding the hand (Fig. 4 (b)), as well as between the area surrounding the hand and the background (Fig. 4 (c)), using the Bhattacharyya distance. Using the colour differences as features is motivated by the idea that when a hand is manipulating an object, distinct colours may be present in the pixels close to the hand.

Motion features used optical flow differences between the hand and its surrounding region, and between the background and the region surrounding the hand. Optical flow differences were quantified as the differences in the histograms of magnitude and direction from a dense optical flow map, using 15 normalized bins. The rationale for using motion contrast is that if an object is manipulated by a hand, the movement velocity and direction in the region around the hand would be similar to the hand region and different from the background.

Hand shape features were obtained by calculating a Histogram of Oriented Gradients (HOG) on the re-sized bounding boxes ($40\times 72$ pixels). $16\times 16$ pixels were used per a cell, and $2\times 2$ cells per block to calculate the HOG for hand shape. To reduce the dimensionality of the HOG, principal component analysis (PCA) was applied to generate 60 principal components as the HOG feature vector.

#### d: Interaction Detection

All 81 annotated tasks were included for interaction detection. A binary random forest classifier with 150 trees was used to predict hand-object interactions.

#### e: Data Post-Processing

Both the predictions and manual annotations were smoothed by a moving average filter with a window of 120 frames (4 seconds). Next, the filtered outputs were normalized by subtracting the minimum value and then dividing by the range of values in each task. The threshold for an interaction was set at 0.5.

#### f: Performance Evaluations

After filtering and normalizing, the average F1 score, precision and recall of leave-one-subject-out (LOSOCV) and leave-one-task-out cross validations (LOTOCV) were calculated to describe the performance of the hand-object interaction detections. In the leave-one-task-out analysis [41], [42], a single task from a single participant was left out for testing; the training set therefore included other tasks from the same participant, and in some cases similar tasks from other participants. Eighteen no-interaction tasks were included in the dataset but could not be used for the LOTOCV F1-score calculations, since they contain no true positives. Thus, accuracy but not F1-score were evaluated for these tasks during LOTOCV. The no-interactions tasks not being left out were included in the training set in all cases.

#### g: Correlation Between F1-Scores and Interaction Percentages

A potential challenge in the evaluation of the classifier is that some individuals with stroke may use their more-affected hands very little, resulting in few true positives, and therefore unreliable estimates of precision and F1-score. To evaluate this potential factor, we also examined the relationship between the F1-score and the interaction percentage by computing the Pearson correlation in SPSS. The interaction percentage was the number of interaction instances over the total number of analyzed hand instances, for a given participant and hand, based on ground truth annotations. An interaction instance is when a hand interacts with an object in a frame. The interaction percentage was used rather than the total number of interaction instances in order to compensate for the different lengths of the videos. Individuals who use their hand very little, and thus are most susceptible to having insufficient evaluation data, are expected to have lower interaction percentages.

### E. Interaction Feature Analysis

In order to evaluate the relative importance of the different feature types, LOTOCV using one feature type at a time was used to analyze features for the interaction classifications. The features with the highest F1-scores in interaction detection were retained for use as well in the subsequent hand role classification analysis.

### F. Hand Role Classification

In addition to hand-object interaction detection, hand role classification was included in order to investigate how functional asymmetry (due to hemiparesis) would affect performance of ADLs among stroke survivors. In addition to the features retained from the interaction detection analysis, hand size change was introduced as an additional feature for hand role classification. The hand role classification analysis was conducted using *bilateral tasks only*, which were manually identified in the labeled videos. Within these tasks, hand size change features were computed in frames where at least one hand was annotated as interacting. This feature consisted of a vector of differences in the number of hand segmentation pixels between consecutive frames over the 10 subsequent frames, normalized by the total number of hand pixels of the current frame. This feature reflects fine movements and was extracted for each hand in order to capture temporal information indicating whether a hand was stabilizing or manipulating an object.

Another binary random forest classifier with 150 trees was used for the hand role classification. The predictions and annotations were also smoothed and filtered with the same method as interaction detections. The threshold to differentiate hand role was set at 0.5. Predictions above threshold were classified as manipulators, or otherwise, as stabilizers. Average F1-score, precision, recall and accuracy during LOSOCV and LOTOCV were reported for the hand role classification performance. As in the interaction detection case, accuracies but not F1-scores were computed for the no-interaction tasks in the LOTOCV hand role analysis.

## III. Results

The demographic information and severity of upper limb impairments for the nine participants are shown in Table 1. Participants had a range of upper limb severity levels according to the total score of FMA-UE [43]: severe (< 25), moderate (between 26 and 50), and mild (>26) upper limb impairment. Some participants had full scores of FMA-UE or ARAT and still self-reported that they had some difficulty with highly skillful tasks, such as writing. Therefore, they fit the criteria of “self-reported impact of affected hand on ADLs” and were included in the study. The resulting dataset consisted of 51,454 frames, including 54% interactions and 46% non-interactions. Each participant had 8 or more tasks across 6 rooms reported in this study. In the dataset, the average total number of instances of more-affected and less-affected hands across participants were 2,499.4 ± 1,641.3 and 3,631.7 ± 2,528.5, respectively.

**TABLE 1:** Demographic Information and Upper Limb Impairment Severity of Participants

Subject ID	Sex	Age (year)	Years from Stroke Onset	FMA-UE total score (out of 66)	ARAT total score (out of 57)	Amount of More-affected Hand Use in MAL ^a^ (score /full score)
1	Male	83	34	27	25	4/145
2	Male	68	4	27	18	8/145
3	Male	66	1	56	57	80/135
4	Female	33	18	24	16	21/150
5	Female	48	7	47	21	40/140
6	Male	41	1	66	57	114.5/135
7	Male	74	3	60	57	32/145
8	Female	63	2	37	26	33.5/150
9	Male	83	34	66	57	145/150
Mean ± SD		60.0 ± 16.1	7.9 ± 11.2	45.6 ± 17.3	37.1 ± 19.1	

FMA-UE: Fugl-Meyer Assessment for Upper Extremity. ARAT: Action Research Arm Test. MAL: Motor Activity Log-30.

^a^ The full score of the MAL is determined by the number of non-N/A items for each individual.

### A. Hand Detection

28,228 frames from 9 stroke survivors, including 19,388 frames with more-affected hands and 22,301 frames with less-affected ones, were manually annotated with hand bounding boxes to evaluate the hand detection accuracy. IoU, F1-score, precision and recall for each hand are shown in Table 2. The average IoU was 0.57 ± 0.06 and 0.69 ± 0.05 for more-affected and less-affected hands, respectively. The average F1 score for more-affected hands and less-affected hands were 0.81 ± 0.25 and 0.85 ± 0.17, respectively. The lower average precision for more-affected hands can be attributed in part to cases where the hands were partially visible at the edge of the frame. As noted above, we did not annotate any bounding boxes for hands that were less than 50% visible, however the hand detector was robust in predict bounding boxes for these incomplete hand regions, which led to false positives. All predicted bounding boxes were used to extract features for hand-object interactions.

**TABLE 2:** Hand Detection Results of More-Affected and Less-Affected Hands

	IoU	F1-score	Precision	Recall
More-affected Hand (N=19,388)	0.57 ± 0.06	0.81 ± 0.25	0.66 ± 0.36	0.99 ± 0.05
Less-affected Hand (N=22,301)	0.69 ± 0.05	0.85 ± 0.17	0.78 ± 0.22	0.98 ± 0.10

IoU: Intersection over Union.

### B. Hand-Object Interaction Detection

The average results of LOSOCV and LOTOCV are shown in Table 4. Overall results included all the instances from both hands. In the overall results, the average F1-score across subjects and tasks were 0.74 ± 0.15 and 0.78 ± 0.18, respectively. The results showed that the algorithm was robust for identifying the hand-object interactions of stroke survivors, without considering whether the hand was more-affected or less-affected.

**TABLE 3:** Feature Subset Average F1-Score and Accuracy of Leave-One-Task-Out-Cross-Validation from 9 Participants

Feature	F1-Score (Mean ± SD)	Accuracy (Mean ± SD)
Optical Flow	0.78 ± 0.18	0.70 ± 0.20
HOG	0.78 ± 0.18	0.70 ± 0.20
HSV	0.77 ± 0.18	0.68 ± 0.20
Optical Flow and HOG	0.78 ± 0.18	0.70 ± 0.19
Optical Flow, HOG, and HSV	0.78 ± 0.18	0.70 ± 0.20

HOG: Histogram of Oriented Gradients

HSV: Hue, Saturation and Value

**TABLE 4:** Hand-Object Interaction Results of Each Participant and Average Across Leave-one-Subject-Out-Cross-Validation, and Leave-One-Task-Out-Cross-Validation

Subject ID	More-affected Hand					Less-affected Hand					Overall				
	$F$	$P$	$R$	$A$	IP	$F$	$P$	$R$	$A$	IP	$F$	$P$	$R$	$A$	IP
1	0.56	0.50	0.63	0.52	45%	0.82	0.71	0.97	0.74	59%	0.71	0.63	0.82	0.63	52%
2	0.31	0.22	0.50	0.86	8%	0.89	0.82	0.99	0.83	67%	0.83	0.73	0.95	0.85	37%
3	0.27	0.17	0.60	0.42	22%	0.48	0.32	1.00	0.42	30%	0.39	0.26	0.85	0.42	26%
4	0.56	0.41	0.86	0.47	40%	0.68	0.97	0.52	0.61	69%	0.62	0.61	0.64	0.54	54%
5	0.81	0.69	0.98	0.69	65%	0.78	0.96	0.66	0.69	76%	0.80	0.79	0.81	0.69	71%
6	0.90	0.83	0.99	0.87	57%	0.53	0.38	0.87	0.69	21%	0.78	0.65	0.97	0.78	39%
7	0.83	0.72	0.99	0.78	55%	0.96	0.94	0.98	0.92	88%	0.90	0.84	0.97	0.84	71%
8	0.60	0.55	0.65	0.58	47%	0.90	0.85	0.95	0.82	81%	0.79	0.74	0.84	0.70	64%
9	0.91	0.85	0.99	0.85	78%	0.43	0.28	1.00	0.85	9%	0.87	0.78	0.98	0.87	44%
Average LOSOCV (Mean ± SD)	0.64 ± 0.24	0.55 ± 0.25	0.80 ± 0.20	0.67 ± 0.18		0.72 ± 0.20	0.69 ± 0.29	0.88 ± 0.17	0.73 ± 0.15		0.74 ± 0.15	0.67 ± 0.17	0.87 ± 0.11	0.70 ± 0.15	
Average LOTOCV (Mean ± SD)	0.76 ± 0.23	0.62 ± 0.36	0.85 ± 0.24	0.68 ± 0.28		0.82 ± 0.22	0.77 ± 0.33	0.87 ± 0.23	0.74 ± 0.29		0.78 ± 0.18	0.74 ± 0.24	0.86 ± 0.20	0.70 ± 0.20	

$F$: F1-score. $P$: Precision. $R$: Recall. A: Accuracy. IP: Interaction Percentage.

LOSOCV: Leave-one-subject-out-cross-validation. LOTOCV: Leave-one-task-out-cross-validation.

**FIGURE 5.**
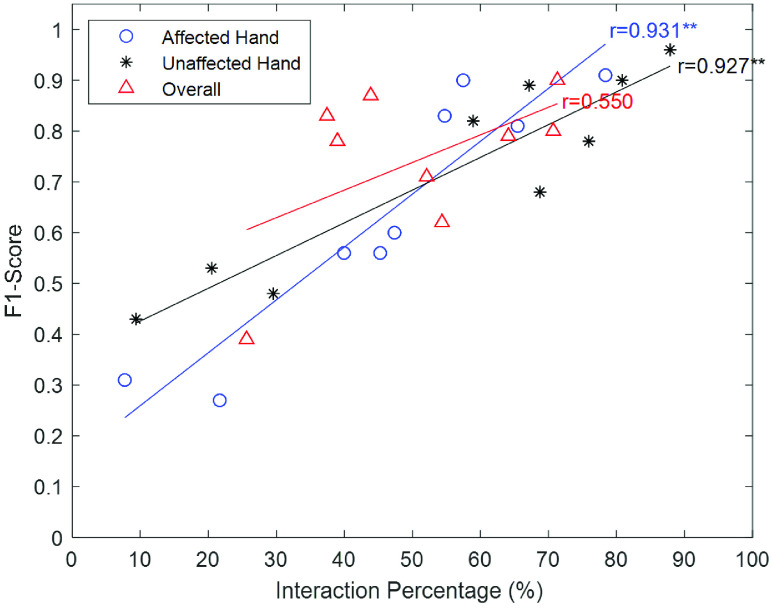
Average F1-scores, interaction percentages and pearson correlations for more-affected hands, less-affected hands, and overall instances of each participant.

For less-affected hands, the average F1-scores across subjects and tasks were 0.72 ± 0.20 and 0.82 ± 0.22, respectively. The average F1-scores for more-affected hands across subjects and tasks were 0.64 ± 0.24 and 0.76 ± 0.23, respectively. The lower F1-scores for more-affected hands might be caused by low more-affected hand interaction percentages. Fig. 5 shows the Pearson correlation results between F1-score and interaction percentage for each participant. Significant correlations were found in both more-affected ($r=0.93$, $p < 0.01^{\ast \ast }$) and less-affected hands ($r=0.93$, $p < 0.01^{\ast \ast }$). The higher the interaction percentage, the higher the F1-score of interaction detection. The correlation for the combined data from both hands was not significant ($r=0.55$, $p>0.05$), which may be attributed to the variations in the number of more-affected and less-affected instances included across participants.

### C. Feature Analysis

The average F1-score and average accuracy of each feature from 9 participants are shown in Table 3. The results showed that optical flow and HOG had the highest F1-scores, which were 0.78, for hand-object interaction detections. Therefore, both optical flow and HOG were subsequently used to classify hand roles.

### D. Hand Role Classification

There were 31,908 frames from 43 bimanual tasks used for the hand role classifications. The bimanual task dataset included 49% manipulator instances and 51% stabilizer instances. Optical flow, HOG, and hand size change features were used to detect hand roles in the samples annotated with hand-object interactions in bimanual ADLs. The average F1-score, precision, recall and accuracy are shown in Table 5. The average F1-score of LOSOCV and LOTOCV for more-affected hands were 0.70 ± 0.19 and 0.68 ± 0.23, respectively. The LOSOCV and LOTOCV F1-score for more-affected hands were both higher than the less-affected ones, which were 0.59 ± 0.23 and 0.65 ± 0.28, respectively. The average accuracies of LOTOCV for more-affected and less-affected hands were 0.66 ± 0.26 and 0.67 ± 0.26, which showed that the algorithm was able to identify hand roles, stabilizers and manipulators, in unseen bimanual tasks for both hands.

**TABLE 5:** Hand Role Classification Results of Each Participant and Average Across Leave-One-Subject-Out-Cross-Validation, and Leave-One-Task-Out-Cross-Validation

Subject ID	More-affected Hand				Less-affected Hand				Overall			
	$F$	$P$	$R$	$A$	$F$	$P$	$R$	$A$	$F$	$P$	$R$	$A$
1	0.46	0.35	0.69	0.32	0.58	0.48	0.71	0.63	0.53	0.41	0.75	0.50
2	0.87	0.77	1.00	0.82	0.41	0.43	0.39	0.34	0.46	0.46	0.47	0.37
3	0.66	0.49	1.00	0.68	0.49	1.00	0.32	0.75	0.44	0.44	0.43	0.68
4	0.54	0.89	0.39	0.46	0.62	0.76	0.52	0.64	0.57	0.82	0.44	0.53
5	0.68	0.81	0.59	0.81	0.80	0.70	0.93	0.72	0.76	0.70	0.84	0.75
6	0.47	0.31	1.00	0.89	0.79	0.66	1.00	0.85	0.35	0.21	1.00	0.69
7	0.93	0.96	0.91	0.88	0.39	0.99	0.24	0.59	0.75	0.94	0.63	0.71
8	0.75	1.00	0.60	0.62	0.25	0.36	0.19	0.54	0.54	0.74	0.43	0.58
9	0.95	0.91	1.00	0.99	0.97	1.00	0.95	0.98	0.95	0.91	1.00	0.99
Average LOSOCV (Mean ± SD)	0.70 ± 0.19	0.72 ± 0.27	0.80 ± 0.23	0.72 ± 0.22	0.59 ± 0.23	0.71 ± 0.25	0.58 ± 0.32	0.67 ± 0.18	0.60 ± 0.19	0.63 ± 0.25	0.67 ± 0.24	0.64 ± 0.18
Average LOTOCV (Mean ± SD)	0.68 ± 0.23	0.83 ± 0.25	0.69 ± 0.29	0.66 ± 0.26	0.65 ± 0.28	0.80 ± 0.31	0.69 ± 0.30	0.67 ± 0.26	0.62 ± 0.24	0.75 ± 0.27	0.64 ± 0.29	0.62 ± 0.27

$F$: F1-score. $P$: Precision. $R$: Recall. $A$: Accuracy.

LOSOCV: Leave-one-subject-out-cross-validation. LOTOCV: Leave-one-task-out-cross-validation.

## IV. Discussion

In this study, LOSOCV and LOTOCV were used to evaluate whether hand-object interactions and hand roles could be automatically identified in egocentric videos of stroke survivors engaging in ADLs in a home simulation laboratory. The results showed that detecting interactions and hand roles of stroke survivors using this type of wearable technology is feasible. The evaluations of interaction and hand roles may serve as a basis for outcome measures that capture the *performance* domain of the ICF, in contrast to the *capacity* domain captured by clinic-based hand function assessments. The trends in our results and possible reasons for them are described below.

**FIGURE 6.**
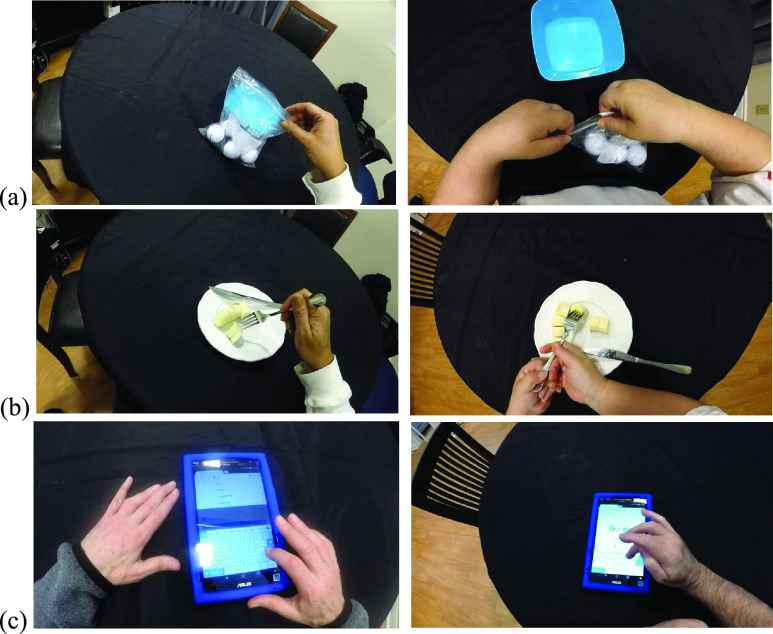
Examples of the same tasks being performed using different strategies. (a) Plastic bag zipping tasks performed by one and both hands. (b) Eating tasks carried out without and with help of less-affected hand. Atypical hand postures of both hands were found when the less-affected (right) hand assisted the more-affected (left) one. (c) Typing tasks performed with different hand postures by two participants with mild upper extremity impairments.

For hand-object interaction, the average F1-score of LOTOCV was higher than LOSOCV, suggesting that including interaction instances from the same participant in the training and testing set led to better predictions. One possible reason is that hand-object interaction instances were diverse among participants, such that including some instances from the same individual in the testing set can help improve predictions. Note that the testing set never contained examples of the same individual performing the same task. In addition, the average LOSOCV F1-score for more-affected hands was only 0.64 ± 0.24, which might have been caused in part by the differences in interacting hand posture across individuals, or by insufficient interaction instances in the training set. These results were obtained despite the fact that the training set contained participants with similar levels of upper extremity impairment as the testing set. The hand postures used during interactions were varied across participants and tasks. To illustrate these variations in hand postures, Fig. 6 provides examples of different strategies being used for the same task. These include using one hand or two hands (Fig. 6 (a)), and using the less-affected hand or not to assist the more-affected one (Fig. 6 (b)). Even a simple task which could be expected to have less scope for variation, such as typing on a tablet, was performed by two participants with similar upper extremity impairment using different postures (Fig. 6 (c)).

In addition to the difference of hand posture, the other factor to impact the average interaction detection F1-score was the interaction percentage. The interaction percentage was determined by participants’ upper extremity impairment levels and whether their more-affected hand was the dominant hand pre-injury. Stroke survivors tend to use their less-affected hands to perform ADLs due to convenience, particularly if their dominant hand remains less-affected. The interaction percentages of more-affected hands tended to be lower than less-affected ones. The low more-affected hand interaction percentage might lead to insufficient more-affected hand interaction instances in the testing samples for certain participants, providing fewer opportunities for true positives and more opportunities for false positives, thus reducing the precision and F1-score, as observed in Fig. 5. Across the entire dataset, this trend can also lead to fewer examples of interacting more-affected hands overall. A potential solution to increase the interaction instances of more-affected hands would be to apply data augmentation techniques such as rotating or re-sizing the images to synthetically generate more instances.

For the identification of hand roles in bimanual ADLs, variations in the size of the hand segmentation were used to reflect finger movements, which are vital to distinguishing manipulation from stabilization actions. Estimating hand size changes from small hand regions and the occlusions of fingers by held objects were the main obstacles to classifying hand roles from egocentric videos. The hand role classification performance was higher for the more-affected hands than less-affected ones. A possible contributing factor for this difference may be found in previous findings suggesting that more-affected hands are used more often as stabilizers [31]. Stabilizers maintain static contact on objects, and their less variable postures might be easier for the algorithms to identify. In contrast, less-affected hand mainly were reported to act as manipulators, which included fast finger movements. Additional features that can capture details of fine finger motions should be explored in the future. Moreover, the hand role results were only based on bimanual ADLs, leading to a smaller number of instances compared with the interaction results.

Previous attempts to capture hand use using wearable technology have focused on capturing body movement, while our approach directly targets *functional* hand use. For example, the Manumeter has been validated primarily for the accuracy of the joint angle estimations [17]. Rowe *et al.* reported the relationship between arm and finger movements during functional tasks [44], but do not provide data on amount of use that could serve as a comparison point for our results. Liu *et al.* and Lee *et al.* explored the use of finger-mounted accelerometers. Their evaluations included amounts of movement, correlations with handedness questionnaires [12], and accuracy in measuring relative movements between the fingers and the wrist [16], but did not directly validate the accuracy of the system for detecting functional hand use. In contrast, the approach proposed here is unique in providing contextual information in addition to body movements, and has been validated directly against ground-truth annotations of functional hand use. Thus, scenarios such as non-functional hand movements and static functional hand use (e.g. using the affected hand to stabilize an object) can be addressed. Conversely, limitations of the approach include greater processing complexity and the fact that some hand-object interactions may be missed if they occur outside the field of view of the camera. Field of view problems did not, however, occur in this study, and it is expected that individuals with hand impairments would only infrequently carry out manipulations without looking at the target object. While the use of video collection in the home is accompanied by privacy considerations, we have previously found the use of egocentric cameras in the home to be feasible [45] and acceptable [46].

Although this study provides a demonstration of feasibility and a baseline for performance, avenues exist to further improve the detection of hand-object interactions and the classification of hand roles from egocentric video after stroke. There were two limitations in this study. The first was the small number of participants. Although the number of hand instances in the egocentric video was sufficient for the purposes of this study, interaction instances were more varied across individuals after stroke compared to healthy individuals. Recruiting more individuals with stroke or augmenting the current dataset may be helpful to enhance the diversity of the training set and enable the classifiers to capture the desired information. The second was that only 3 feature types were used to detect interactions and hand roles. The features demonstrated feasibility and were based on a priori insights about the problem and prior success in similar problems [30], but might not be sufficient to capture complex hand movements, especially among stroke survivors. A transition from manually engineered features to deep learning may also be beneficial to extract more details of finger movements and to identify the interactions and hand roles of stroke survivors, but will require greater amounts of data. The use of a home simulation lab can be considered an additional limitation, however our dataset is expected to be reflective of real home conditions because of the variety in tasks and objects across 6 different room settings.

We expect that this study can benefit clinicians and researchers by improving our understanding of the hand use of stroke survivors during ADLs at home, and by providing valuable information that is not accessible in the clinic. Frame-by-frame interaction detection results could be used as the basis for new outcome measures reflecting the number, frequency, or duration of functional interactions at home over time, and possibly capturing aspects of the ICF performance domain not reliably captured by current outcome measures. Further, the information could be used to develop individualized rehabilitation plans for stroke survivors. While our focus here was on detecting the amount of functional hand use, egocentric video is a platform that can be used for additional applications, such as postural analysis and recognition of specific activities.

## V. Conclusion

A wearable system based on egocentric video and machine learning can provide the basis for quantifying hand use outside of clinical environments after stroke, as well as the role of the more-affected hand in bimanual interactions. The algorithms were able to detect interactions and hand roles in unseen subjects and tasks. By capturing home ADLs with this wearable system, the performance component of hand function can be quantified and used to evaluate and guide therapeutic plans for stroke survivors. Including some subject-specific annotations in the training samples may increase the accuracies of interaction and hand role predictions. Larger datasets, additional features and deep learning approaches should be explored to improve upon the feasibility demonstration provided by this study.
